# Recommendations for management of pregnancy complicated by Caroli disease: A case report and literature review

**DOI:** 10.1016/j.iliver.2025.100199

**Published:** 2025-11-04

**Authors:** Chen Liang, Li Bai, Wei Hou, Wen-Yan Song, Yun-Xia Zhu, Su-Jun Zheng

**Affiliations:** a Department of Liver Disease, Beijing You'an Hospital, Capital Medical University, Beijing 100069, China; b Beijing Municipal Key Laboratory of Liver Failure and Artificial Liver Treatment Research, Beijing 100069, China; c Department of Gastroenterology, Beijing Jishuitan Hospital, Capital Medical University, Beijing 100035, China; d Department of Medical Imaging, Beijing You'an Hospital, Capital Medical University, Beijing 100069, China; e Department of Obstetrics and Gynecology, Beijing You'an Hospital, Capital Medical University, Beijing 100069, China

**Keywords:** Caroli disease, Pregnancy, Multidisciplinary, Management, Recommendation

## Abstract

We investigated the clinical and genetic characteristics of a 28-year-old pregnant Chinese woman with Caroli disease (CD) and systematically reviewed the published literature on pregnancy complicated by CD. We reviewed our case and 8 patients with 9 pregnancies included in 6 reports. The main clinical characteristics of pregnancy complicated by CD are cholestasis, liver and renal insufficiency, portal hypertension, and cirrhosis. In severe cases, there may be an increased risk of disease progression during pregnancy. Management of pregnancy complicated by CD is primarily supportive. Antimicrobial therapy and prevention of cholestasis and portal hypertension are recommended. Patients may benefit from multidisciplinary care.

## 1 Case presentation

**Fig. 1:**
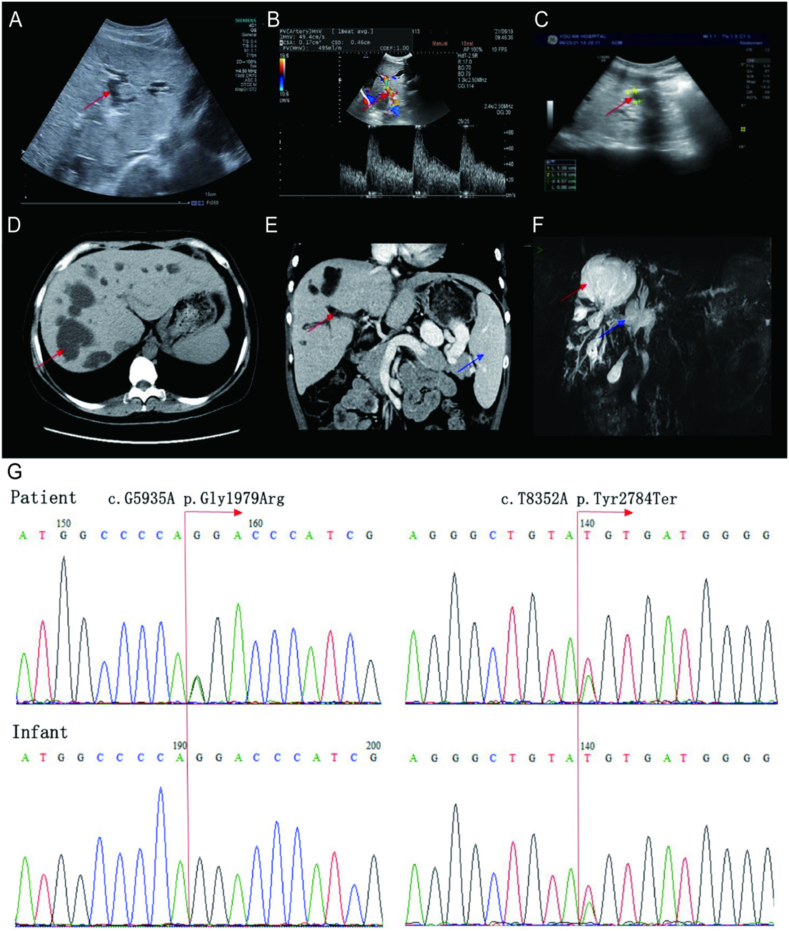
Findings of the imaging examinations. Ultrasonography showed (A) cystic dilatation of the intrahepatic bile ducts (red arrow), (B) occlusion of the main portal vein and its branches (red arrow), and (C) a renal cyst (red arrow). Contrast-enhanced computed tomography revealed (D) a hepatic cyst (red arrow) and (E) splenomegaly (blue arrow). Magnetic resonance cholangiopancreatography revealed (F) cystic dilatation of the intrahepatic bile ducts (red arrow) and a hepatic cyst (blue arrow). (G) Sanger sequencing revealed two mutations in *PKHD1*, c.G5935A(p.Gly1979Arg) and c.T8352A(p.Tyr2784Ter).

The patient was a 28-year-old pregnant Chinese woman who was admitted to Beijing You'an Hospital at 32 weeks' gestation after a 20-week history of elevated alkaline phosphatase (ALP) and total bile acid (TBA). She had also experienced two episodes of fever and had elevated urinary protein and leukocytes. Previous treatment had included ursodeoxycholic acid (UDCA) for cholestasis and empirical antibiotic therapy for suspected infection (regimen not specified). The main laboratory results upon admission are shown in Table 1. Urine culture revealed urinary protein (+), urinary leukocytes (+), and *Escherichia coli*. Serum bilirubin, anti-cardiolipin antibody, autoantibodies, and immunoglobulin levels were normal. Abdominal ultrasonography revealed multiple cysts in the liver and kidneys, cystic dilatation of the intrahepatic bile ducts, thrombosis of the main portal vein and its branches, splenomegaly, and a collateral circulation. Whole-exome sequencing was performed to investigate the genetic characteristics of her CD. A novel compound heterozygous mutation consisting of c.G5935A (p.Gly1979Arg) and c.T8352A (p.Tyr2784Ter) was found in *PKHD1,* the polycystic kidney and hepatic disease 1 gene (Fig. 1, with prediction of pathogenicity shown in Table S1). The same heterozygous mutation (c.T8352A) was found in the male newborn (Fig. 1).

**Table 1:** Main laboratory findings for the patient.

Examination item	Normal range	21.6.2021	24.6.2021	28.6.2021	30.6.2021	03.08.2021	31.08.2021	06.08.2021	11.04.2022
Alkaline Phosphatase(U/L)	35–100	104.0	83.0	76.0	67.0	65.0	56.0	116.0	126.0
γ-Glutamyl transpeptidase(U/L)	7–45	19.0	17.0	14.0	18.0	19.0	16.0	29.0	35.0
Total serum bile acid (μmol/L)	<10	28.7	21.4	22.9	12.0	15.4	51	23	8.9
Total bilirubin (μmol/L)	5–12	10.0	13.3	10.6	9.4	21.3	18.5	20.2	15.8
Direct bilirubin (μmol/L)	<7	2.6	3.4	2.9	2.6	4.9	4.8	5.6	4.2
Creatinine (μmol/L)	41–73	107	101	98	98	106	103	75	92
Estimated glomerular filtration rate (mL/min/1.73 m^2^)	>90	60.8	65.2	67.6	67.6	61.5	63.7	92.8	72.5
White blood cell (× 10^9^/L)	3.5–9.5	13.27	5.85	3.04	2.90	4.16	3.22	3.77	3.26
Hemoglobin (g/L)	115–150	92	77	80	77	108	104	98	97
Platelets (× 10^9^/L)	125–350	100	85	84	86	121	97	127	157
Neutrophil count (× 10^9^/L)	1.8–6.3	12.62	4.81	2.04	1.86	2.79	2.19	2.76	2.29
D-dimer (μg/L)	<230	322.0	394.0	447.0	–	81.0	–	–	–
C-reactive protein (mg/L)	<10	8.03	65.66	4.06	–	–	–	–	–

- Indicating that this examination was not reviewed at this time point.

Based on the clinical, laboratory, and imaging data and the compound heterozygous *PKHD1* mutation, the patient was diagnosed to have Caroli disease (CD)^1^ and referred for multidisciplinary management. She was started on UDCA, low-molecular-weight heparin, and ceftriaxone [(FDA pregnancy risk category B), after which her ALP and TBA levels decreased, her estimated glomerular filtration rate (eGFR) improved, and her urinary leukocytes resolved. Cesarean section was performed at 32 weeks + 5 days of gestation. Abdominal ultrasonography and laboratory tests did not identify any relevant characteristics of CD in the newborn.

CT findings post-delivery provided further evidence in support of a diagnosis of CD. Magnetic resonance cholangiopancreatography revealed dilatation of the intrahepatic bile ducts (Fig. 1). Gastroscopy showed reflux esophagitis and chronic superficial gastritis.

The patient and her infant remain under regular telephone follow-up and are well. There are no family members with any CD-related signs or symptoms.

## 2 Literature review and discussion

CD is a rare autosomal recessive disorder. The mutations of the *PKHD1* gene is considered as the major contributor, which leads to the functional defect of fibrocystin protein and affects the vital receptors in the protein signaling pathway regulating bile acid synthesis, thus resulting in cholestasis, cholangitis, cystic dilatation of intrahepatic ducts and ensuing structural damage of the liver parenchyma. *PKHD1* is expressed primarily in the kidney, so CD is often accompanied by autosomal recessive polycystic kidney disease.^1^ CD affects women of childbearing age, in whom chronic liver or kidney disease is a risk factor for adverse antenatal and perinatal outcomes.^2^^,^^3^ Moreover, CD may worsen in some patients, leading to fulminant liver or kidney failure. However, there is limited information available on this condition.^4^, ^5^, ^6^, ^7^, ^8^, ^9^ Therefore, we undertook a literature review on pregnancy complicated by CD in the hope of improving management for this patient group.

We searched the PubMed, Cochrane Library, and Embase databases from inception to August 2025 (Fig. S1) using a series of search terms (see File S2). Eight patients (including our case) with 9 pregnancies were identified (Table S2).^4^, ^5^, ^6^, ^7^, ^8^, ^9^ These patients usually presented with a combination of cholestasis, hepatic or renal insufficiency, cysts in the liver and kidney, portal hypertension, and cirrhosis. Biliary dilatation caused recurrent cholangitis. Pregnant women were at higher risk of urinary tract infection and cholecystitis. Cholangitis and urinary tract infections during pregnancy can increase the risks of spontaneous abortion, premature birth, and perinatal mortality. Gram-negative sepsis is a leading cause of death in these patients. The well-known increases in blood volume and flow during pregnancy are mediated by increased aldosterone. In this regard, the risk of variceal bleeding seems to be significantly increased in CD with portal hypertension during pregnancy. Preeclampsia, transient or permanent deterioration of liver or kidney function, disseminated intravascular coagulation, and even acute respiratory distress syndrome have been documented in severe cases during pregnancy. Patients with more severe CD may be at higher risk of disease progression during pregnancy. We also noted that kidney function was poorer in patients with four or more pregnancies than in those with fewer pregnancies. The optimal gestational week and mode of delivery for women with CD is unclear. However, some researchers have recommended delivery at 37 weeks or earlier to decrease the risk of late stillbirth.

Our patient presented with infection, elevated ALP and TBA levels, and a decreased eGFR, but with blood pressure within the normal range. It is difficult to determine whether pregnancy accelerated disease progression in this patient because her organ status pre-pregnancy was unknown. However, her serum TBA and creatinine levels were lower and her eGFR was higher after delivery than during pregnancy. Therefore, in this case, it is unlikely that pregnancy would have accelerated liver and renal dysfunction in the long term. Cesarean section was performed at 32 weeks + 5 days in view of the risks of liver failure and gastrointestinal hemorrhage as a result of thrombosis of the portal vein and portal hypertension. The patient and her newborn recovered well after delivery.

This patient has a compound heterozygous mutation in *PKHD1* (p.Gly1979Arg/p.Tyr2784Ter) and her infant has a single mutation (p.Tyr2784Ter). The mutation detected in the infant results in defunctionalization of the protein fibrocystin. Therefore, the infant requires long-term monitoring for CD.

The present guidelines do not include recommendations for pregnancy complicated by CD.^1^ However, based on our research, we propose the following management strategy. ALP, gamma-glutamyl transferase, TBA, and eGFR should be monitored closely. Supportive and symptomatic treatment is essential, including dietary and fluid intake restrictions, treatment for anemia, and pancytopenia. Antibiotic treatment should be used for biliary tract or kidney infection. The selection of antibiotics should be guided by the FDA pregnancy risk category.^10^ Patients with CD should receive UDCA even if asymptomatic. UDCA has been shown to reduce the incidence of preterm birth, cardiac dysfunction in the fetus, respiratory distress syndrome, and admissions to the neonatal intensive care unit.^11^^,^^12^ Assistance with fertility management and delivery instructions are necessary. The optimal gestational week and delivery mode should be chosen. All patients should be informed of the possible complications and that adverse consequences during pregnancy can be prevented if carefully managed.

Multidisciplinary management is essential in pregnant women with CD in view of the broad range of symptoms and complications of the disease as well as the particularity of maternal and infant care. The multidisciplinary team should include hepatologists, gastroenterologists, obstetricians, neonatologists, geneticists, radiologists, and psychologists. Monitoring and follow-up plans should be individualized to the patient. Routine examinations should be performed. Hepatologists and gastroenterologists can help with prevention and treatment of symptoms or complications related to CD. Obstetricians can assist in determining the optimal gestational week and delivery mode. Newborns should be evaluated and followed up for CD. Radiologists can make a prenatal diagnosis of CD and geneticists can provide genetic counseling. Patients with CD can opt for assisted reproductive technology and have healthy children. These women should also be observed for anxiety about disease progression and fear of giving birth to a child with CD.

## 3 Summary

We have documented the clinical manifestations and course in a pregnant woman with CD who was found to have a compound heterozygous mutation (p.Gly1979Arg/p.Tyr2784Ter) in *PKHD1*. Management of such cases is primarily supportive and symptomatic. These patients would benefit from multidisciplinary management.

## CRediT authorship contribution statement

**Chen Liang:** Writing – review & editing, Writing – original draft. **Li Bai:** Writing – review & editing, Writing – original draft. **Wei Hou:** Writing – original draft, Writing – review & editing. **Wen-Yan Song:** Data curation, Supervision. **Yun-Xia Zhu:** Conceptualization, Data curation, Supervision. **Su-Jun Zheng:** Supervision, Funding acquisition, Conceptualization.

## Informed consent

Written informed consent for publication of this report was obtained from the patient and legal guardian of the infant.

## Ethics statement

All procedures performed in the course of this research were in line with the principles of the Declaration of Helsinki and the ethical standards set down by the Ethical Committee of Beijing You'an Hospital, Capital Medical University, Beijing, China (ethics approval number LL-2019-029-K).

## Data availability statement

The original contributions presented in this research are included in the article/Supplementary Material. Further inquiries can be directed to the corresponding authors.

## Declaration of generative AI and AI-assisted technologies in the writing process

Not applicable.

## Funding

This research was supported financially by the 10.13039/501100009601Beijing Municipal Administration of Hospitals Clinical Medicine Development of Special Funding Support (ZYLX202125), the Key Research Project of 10.13039/100007831Capital Health Development (2022-1-2182), the High-Level Public 10.13039/100018696Health Technical Talents of 10.13039/501100005088Beijing Municipal Health Commission (Academic Leader-02-14), and the 10.13039/501100012166National Key Research and Development Program of 10.13039/100007225Ministry of Science and Technology (2022YFC2304400).

## Declaration of competing interests

The authors and funders have no conflict of interests related to this publication.
