# Supplementary material for: Recommendations for management of pregnancy complicated by Caroli disease: A case report and literature review
Source: ILIVER. 2025 Nov 4;4(4):100199. doi: 10.1016/j.iliver.2025.100199 (PMC12664362; doi:10.1016/j.iliver.2025.100199)
Supplement: Multimedia component 1 [file mmc1.doc]

**
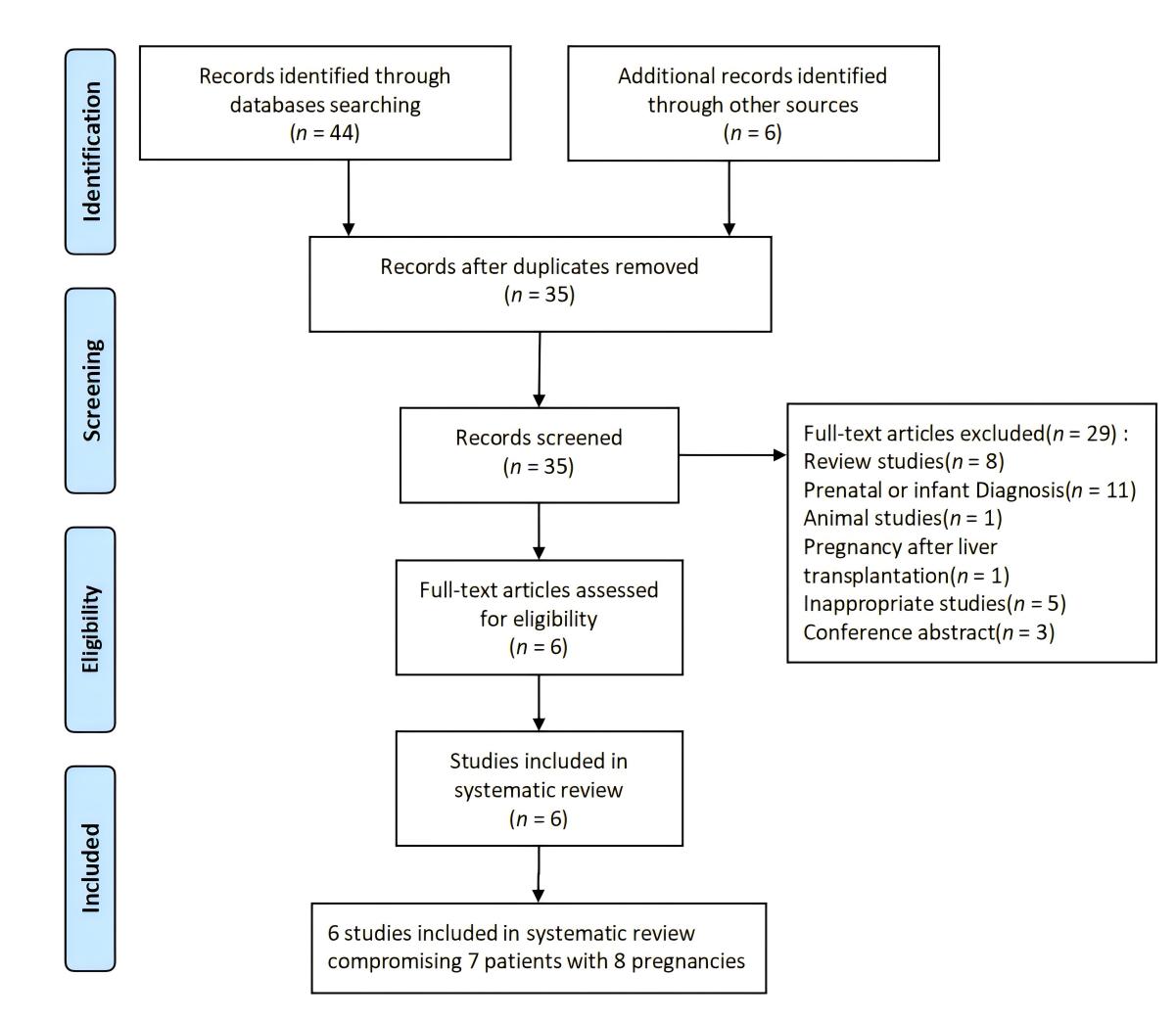
**

**Fig. S1** Flow diagram showing the procedure used for literature screening and selection of relevant reports.
