# Supplementary material for: Recommendations for management of pregnancy complicated by Caroli disease: A case report and literature review
Source: ILIVER. 2025 Nov 4;4(4):100199. doi: 10.1016/j.iliver.2025.100199 (PMC12664362; doi:10.1016/j.iliver.2025.100199)
Supplement: Multimedia component 2 [file mmc2.doc]

**Pubmed**

search terms

MeSH Terms：Caroli Disease

Entry Terms：Disease, Caroli

Caroli's Disease

Carolis Disease

Disease, Caroli's

Caroli's Syndrome

Caroli Syndrome

Carolis Syndrome

Syndrome, Caroli's

MeSH Terms：Pregnancy

Entry Terms：Pregnancies

Gestation

.......................................................

1. " Caroli Disease"[Mesh] 403
2. "disease caroli"[Title/Abstract] OR "caroli's disease"[Title/Abstract] OR "carolis disease"[Title/Abstract] OR "disease caroli's"[Title/Abstract] OR "caroli's syndrome"[Title/Abstract] OR "caroli syndrome"[Title/Abstract] OR "carolis syndrome"[Title/Abstract] OR "syndrome caroli's "[Title/Abstract] 711
3. #1 OR #2 833
4. "pregnancy "[Mesh] 969,316
5. "Pregnancies"[Title/Abstract] OR "Gestation"[Title/Abstract] 206,821
6. #4 OR #5 1,020,543
7. #3 AND #6 22

**Cochrane Library**

search terms

MeSH Terms：Caroli Disease

Entry Terms：Caroli's Syndrome

Carolis Disease

Carolis Syndrome

Caroli Syndrome

Caroli's Disease

Syndrome, Caroli's

Disease, Caroli

Disease, Caroli's

MeSH Terms：Pregnancy

Entry Terms：Pregnancies

Gestation

.......................................................

#1 (Caroli Disease):ti,ab,kw OR (Caroli's Syndrome):ti,ab,kw OR (Carolis Disease):ti,ab,kw OR (Carolis Syndrome):ti,ab,kw OR (Caroli Syndrome):ti,ab,kw (Word variations have been searched) 3

#2 (Caroli's Disease):ti,ab,kw OR (Syndrome, Caroli's):ti,ab,kw OR (Disease, Caroli):ti,ab,kw OR (Disease, Caroli's):ti,ab,kw (Word variations have been searched) 3

#3 #1 OR #2 3

#4 (Pregnancy):ti,ab,kw OR (Pregnancies):ti,ab,kw OR (Gestation):ti,ab,kw (Word variations have been searched) 76010

#5 #3 AND #4 0

**Embase**

search terms

MeSH Terms：Caroli disease

Entry Terms：caroli syndrome

Caroli's disease

carolis disease

disease, caroli

MeSH Terms：Pregnancy

Entry Terms：child bearing

Childbearing

Gestation

Gravidity

intrauterine pregnancy

labor presentation

labour presentation

pregnancy maintenance

pregnancy trimesters

.......................................................

#3. ('caroli disease'/exp OR 'caroli syndrome':ab,ti 22 22 Jun 2022

OR 'carolis disease':ab,ti OR 'disease,

caroli':ab,ti) AND ('pregnancy'/exp OR 'child

bearing':ab,ti OR childbearing:ab,ti OR

gestation:ab,ti OR gravidity:ab,ti OR

'intrauterine pregnancy':ab,ti OR 'labor

presentation':ab,ti OR 'labour

presentation':ab,ti OR 'pregnancy

maintenance':ab,ti OR 'pregnancy

trimesters':ab,ti)

#2. 'pregnancy'/exp OR 'child bearing':ab,ti OR 922,814 22 Jun 2022

childbearing:ab,ti OR gestation:ab,ti OR

gravidity:ab,ti OR 'intrauterine pregnancy':ab,ti

OR 'labor presentation':ab,ti OR 'labour

presentation':ab,ti OR 'pregnancy

maintenance':ab,ti OR 'pregnancy

trimesters':ab,ti

#1. 'caroli disease'/exp OR 'caroli syndrome':ab,ti 1,278 22 Jun 2022

OR 'carolis disease':ab,ti OR 'disease,

caroli':ab,ti
