# Supplementary material for: Recommendations for management of pregnancy complicated by Caroli disease: A case report and literature review
Source: ILIVER. 2025 Nov 4;4(4):100199. doi: 10.1016/j.iliver.2025.100199 (PMC12664362; doi:10.1016/j.iliver.2025.100199)
Supplement: Multimedia component 3 [file mmc3.docx]

**Table S1** Pathogenicity analysis of mutation sites in the *PKHD1* gene.

| Mutation | Change of nucleic acids/amino acids | Polyphen2 | | SIFT | | PROVEAN | | Mutation Taster | | FATHMM | | InterVar | ACMG grade |
| --- | --- | --- | --- | --- | --- | --- | --- | --- | --- | --- | --- | --- | --- |
|  |  | Score | Prediction | Score | Prediction | Score | Prediction | Score | Prediction | Score | Prediction | Prediction |  |
| PKHD1  NM_138694.4  chr6:51934296 | exon37  c.G5935A  p.Gly1979Arg | 1 | probably  damaging | 0.005 | damaging | −3.57 | deleterious | 0.998 | disease  causing | −2.23 | damaging | likely  pathagetic | Possibly pathogenic |
| PKHD1  NM_138694.4  chr6:51791324 | exon53  c.T8352A  p.Tyr2784Ter | - | - | - | - | - | - | 1 | disease  causing | - | - | - | Pathogenic |

**Table S2** Systematic review on Caroli’s disease complicating pregnancy.

| Study | Maternal age  (history of gestation) | Age at onset | Clinical characteristics | | Maternal outcome | Complications during pregnancy | Treatment during illness | Neonatal  condition |
| --- | --- | --- | --- | --- | --- | --- | --- | --- |
|  |  |  | Hepatic | Extrahepatic |  |  |  |  |
| Tsunoda M^2^ | 19y  (1-1-0-0) | 5y | Abdominal mass | Bilateral polycystic kidney;  Fever | Gave birth vaginally at 37gw | Anemia; Thrombocytopenia; Chronic renal failure | Diet control;  Erythropoietin;  Prophylactic oral antibiotics | Partus matures; A 2450 g male infant; Apgar score 9/10 |
| Adaie C^5^ | 28y  (5-0-3-2) | NR | Biliary obstruction; Elevated liver enzymes; Abdomina pain;  Vomiting | Fever;  Acute pyelonephritis;  Pancytopenia | Cesarean section; Intubated | DIC; ARDS | Antibiotic therapy; Cholecystectomy; Choledochectomy; Jejunostomy; Blood and plasma transfusion; Trachea intubation | Prematurity;  No evidence of neonatal CD |
| Banks N^6^  Case3 | NR  (NR) | 23y | Abdominal pain | NR | NR | NR | NR | NR |
| Banks N^6^  Case4 | 33y  (1-1-0-0) | 46y | Splenomegaly;  Liver cysts | Pancytopenia | Cesarean section at 39 gw+4 | NR | Kidney-liver double transplantation | Partus matures;  Birth weight 2863 g |
| Levin G^7^ | 27y  (NR) | 12y | Abdominal pain; End-stage liver cirrhosis; Hepatosplenomegaly; Portal hypertension | Fatigue;  Anemia; | Cesarean section at 29gw; | Dilatation of the blood vessel in the uterine serosa; Hematochezia;  Hypotension; | Beta-blockers | Prematurity;  A 1200 g infant with normal Apgar Scores |
| Yao X^8^ | 26y  (1-0-0-1) | 26y | Hepatomegaly; Splenauxe;  Intrahepatic cystic lesions | Pancytopenia;  Increased creatinine; Tortuous dilated bile duct | Cesarean section at 36gw | NR | Iron sucrose; | Prematurity;  A PKHD1 carrier with one heterozygous  mutations c.G2854A |
| Félix N^9^ | 37y  (1-0-0-1) | 22y | Cholestasis;  Esophageal varices; Cirrhosis;  Elevated hepatic transaminases | Thrombocytopenia; Pruritus; | Gave birth vaginally at 30gw+4 | Increased total bilirubin and worsening of liver function tests; Pruritus and sporadic contractions; Mild jaundice and elangiectasia | Right hepatic lobectomy; Cholecystectomy;  Ursodeoxycholic acid; | Prematurity; Remains well with no apparent sequelae |
| Our study | 20y  (1-0-0-1) | 28y | Splenomegaly;  Cholestasis | Diffuse renal parenchymal lesions;  Anemia;  Thrombocytopenia;  Fever | Cesarean section at 32gw+5d | Acute pyelonephritis;  Thrombus of portal vein;  Fever | Antibiotic;  Low-molecular-heparin;  UDCA | Prematurity;  A PKHD1 carrier with one heterozygous mutations c.T8352A |
|  |  |  |  |  |  |  |  |  |

Abbreviations: (number-number-number-number): (Gestation-Term-Abortion-Preterm); NR: not report; gw: gestational weeks; d: day; m: month; y: year; DIC: Disseminated Intravascular Coagulation; ARDS: Acute Respiratory Distress Syndrome; UDCA: ursodeoxycholic acid.
